# Strengthening Safeguards in the Assisted Dying Bill: A Comparative Review of Ethical, Legal, and Medical Considerations in End-of-Life Legislation

**DOI:** 10.7759/cureus.80572

**Published:** 2025-03-14

**Authors:** Russell Tolentino

**Affiliations:** 1 Edinburgh Law School, The University of Edinburgh, Edinburgh, GBR; 2 Centre for Evidence-Based Medicine, University of Oxford, Oxford, GBR; 3 National Centre for Epidemiology and Population Health, Australian National University, Canberra, AUS

**Keywords:** assisted dying legislation, comparative policy analysis, conscientious objection law, end-of-life care ethics, ethical dilemmas in healthcare, legal safeguards in euthanasia, medical capacity assessment, palliative care regulatory frameworks, patient autonomy, physician-assisted dying

## Abstract

The UK's Assisted Dying Bill aims to give terminally ill individuals the option to choose the timing and manner of their death. This proposal has sparked intense debates regarding the ethical, legal, and medical implications of protecting the rights of terminally ill persons. However, the bill faces considerable challenges in the UK Parliament due to various concerns about its provisions. A critical review of the Assisted Dying Bill reveals key shortcomings in medical assessments, eligibility criteria, conscientious objection, and safeguards against potential abuse. A clearer picture of the necessary improvements has emerged by benchmarking these issues against the more successful assisted dying frameworks in jurisdictions like the US state of Oregon, Canada, the Australian states of Victoria and Western Australia, Belgium, Switzerland, and the Netherlands.

To address these shortcomings, recommendations include enhancing the involvement of specialist physicians, tightening residency requirements, increasing the number of requests for assisted dying, clarifying guidelines for administering lethal medications, mandating the reporting of procedural breaches, and implementing strict measures concerning conscientious objection to safeguard healthcare practitioners. This review aspires to recommend a comprehensive legal framework that permits terminally ill individuals to make informed and voluntary end-of-life decisions while protecting healthcare practitioners from ethical and legal dilemmas, ensuring that any proposed assisted dying legislation embodies a compassionate and ethically sound approach.

## Introduction and background

The Assisted Dying Bill in the UK, formally known as the Terminally Ill Adults (End of Life) Bill, is currently under the consideration of the UK Parliament and provides terminally ill individuals with the right to end their lives through a regulated process of assisted dying [1]. This bill outlines that to be eligible, an individual must be a terminally ill adult with a prognosis of six months or less to live and must have the mental capacity to make an informed decision [1]. The procedure will involve strict safeguards, including multiple assessments by independent doctors and a waiting period to ensure that the decision is voluntary and carefully considered. Currently, assisted dying is illegal in the UK, which has led some individuals to seek euthanasia or assisted suicide in countries like Switzerland, where these practices are legally permitted [2-3]. This bill arises from a growing public demand for legal access to assisted dying services and from ethical concerns regarding the inequity of these services being available only to those who can afford to travel abroad.

Assisted dying refers to a legal process in which a terminally ill individual, after meeting specific eligibility criteria, self-administers prescribed life-ending medication. In contrast, euthanasia involves a third party, typically a medical professional, actively ending a person's life. Assisted suicide allows an individual to self-administer lethal medication with assistance from another person who does not directly cause death. The distinctions among these practices carry significant legal and ethical implications, particularly in shaping the debate surrounding the scope and safeguards of the Assisted Dying Bill.

Despite substantial public support, the bill has faced significant opposition in the UK Parliament due to concerns about coercion, undue influence, and the psychological burden on healthcare practitioners involved in the process. Critics argue that the bill's safeguards may be insufficient to protect vulnerable individuals from external pressure or exploitation, particularly those who might feel compelled to opt for assisted dying due to familial or societal influences [4]. Moreover, opponents of the bill highlight the ethical dilemmas faced by healthcare professionals who may experience moral conflicts when asked to facilitate assisted dying [5].

To address these concerns, the bill proposes a structured legal framework for assisted dying, which includes capacity assessments, multiple formal requests, and the management of lethal medication. However, significant uncertainties remain regarding how voluntary decisions will be verified, whether healthcare practitioners can conscientiously object, and how medication will be dispensed and monitored to prevent misuse.

The review evaluates the Assisted Dying Bill, focusing on the susceptibility of individuals to coercive influences that may lead terminally ill persons to choose assisted dying, as well as the role of healthcare practitioners in this decision-making process. It highlights the lack of strong mechanisms to guarantee that such choices are genuinely voluntary, raising concerns about conscientious objection, capacity assessments, and the management of lethal medication. By comparing the bill to similar legislation in Oregon, Canada, Australia, Belgium, Switzerland, and the Netherlands, the review analyses the effectiveness of safeguards and eligibility criteria, aiming to identify international best practices while considering the ethical, legal, and medical implications of assisted dying. Furthermore, it offers actionable recommendations to enhance the bill and to ensure better protection for terminally ill persons and healthcare practitioners and the integrity of the assisted dying process.

This review was presented to the faculty of Edinburgh Law School, The University of Edinburgh, as a partial fulfilment of the requirements for the degree of Master of Laws in Medical Law and Ethics and archived in the university's digital repository [6].

## Review

Inadequacies in medical assessment and capacity determination

A critical examination of the Assisted Dying Bill reveals substantial deficiencies in its protective measures, particularly with the medical assessment process and capacity determination. The current provisions permit any registered medical practitioner to diagnose a terminal illness and evaluate a patient's decision-making capacity concerning assisted dying. However, this broad criterion lacks the necessary safeguards to ensure such evaluations' accuracy, reliability, and ethical integrity. The absence of mandated involvement from specialists, such as consultant palliative care physicians, psychiatrists, or geriatricians, raises significant concerns about the potential for misdiagnosis, inappropriate eligibility determinations, and flawed capacity assessments.

Medical practitioners without specialised training in end-of-life care, mental health, or complex capacity assessments may not possess the requisite expertise needed to navigate the nuanced clinical, psychological, and ethical dimensions of decisions surrounding assisted dying. The determination of terminal illness presents inherent challenges, particularly given the well-documented prognostic uncertainties in medicine. Misdiagnosis or inaccurate prognostication could lead to unwarranted approvals for assisted dying, especially in instances involving chronic illnesses, neurodegenerative conditions, or psychiatric comorbidities. Furthermore, cognitive impairments, depressive disorders, and various external pressures, whether familial, societal, or financial, may unduly influence a patient's capacity for decision-making. In the absence of psychiatric evaluations or thorough competency assessments, there is an increased risk of endorsing assisted dying requests from individuals whose judgment may be compromised by treatable conditions, such as depression, anxiety, or executive dysfunction.

A foundational principle of the bill is the respect for patient autonomy, ensuring that terminally ill individuals retain the right to make informed choices regarding the timing and manner of their death [7]. However, this autonomy is meaningful only when patients can make decisions free from coercion, undue influence, or psychological distress [8]. The lack of stringent safeguards heightens the risk that patients, particularly those who are elderly, have disabilities, or experience social isolation, may feel pressured to seek assisted dying due to external factors rather than through authentic and autonomous decision-making. Coercion, whether overt or subtle, can prove difficult to detect without comprehensive psychological and social assessments.

Comparative analyses of international assisted dying frameworks indicate stronger protections in jurisdictions such as Oregon, Canada, and the Netherlands [9]. For instance, Oregon's Death with Dignity Act stipulates that two independent physicians must confirm a terminal diagnosis and assess a patient's capacity prior to approving a request for assisted dying [10]. Similarly, Canada mandates additional oversight, including psychiatric evaluations, in cases where uncertainty exists regarding a patient's decision-making capacity. Certain jurisdictions also incorporate legal competence tests to ensure that cognitive impairments do not compromise the integrity of consent [11].

In order to enhance the Assisted Dying Bill and mitigate the risks of misdiagnosis, coercion, and inadequate capacity determinations, it is imperative to consider the implementation of several safeguards. These could include mandatory specialist involvement to ensure accurate prognostic assessments and capacity evaluations, independent second opinions from multiple medical professionals, and introducing standardised legal and medical competency tests to evaluate cognitive function and decision-making abilities. Furthermore, mandating mental health assessments in cases where depression, anxiety, or cognitive impairment may influence the patient's request, along with establishing a systematic review mechanism to evaluate the long-term impact and ethical integrity of assisted dying cases, would significantly strengthen the bill.

Insufficiency of the two-request system

The current Assisted Dying Bill mandates that individuals with terminal illnesses must make two formal requests prior to proceeding with assisted dying. While this provision introduces a degree of deliberation, it arguably lacks sufficient opportunities for reconsideration, particularly in circumstances where individuals may be experiencing fluctuating emotions, external pressures, or transient psychological distress [12]. Comparative analyses with the Australian jurisdictions of Victoria and Western Australia suggest that requiring a minimum of three separate requests could enhance procedural safeguards, ensuring that the decision-making process is both well-considered and free from undue influence [13].

The limitations inherent in the UK's two-request framework warrant consideration. Firstly, the insufficient time for reflection associated with terminal illnesses often entails emotional distress, existential anxiety, and periods of varying mental clarity. Moreover, the impact of fluctuating symptoms and mental states cannot be overlooked, as many terminal conditions are characterised by phases of worsening and ameliorating symptoms, pain, or mood. A two-request process might allow patients to proceed with assisted dying during a temporary crisis rather than after a sustained and considered reflection. A lengthier request process ensures that these decisions are not driven by short-term emotional responses but rather by a consistent and sustained intent.

As exemplified by Victoria's Voluntary Assisted Dying Act of 2017 and Western Australia's Voluntary Assisted Dying Act of 2019, international best practices mandate a minimum of three separate requests [14]. The first request signifies the patient's initiation of the process through a formal verbal request to a medical practitioner. Following a mandatory reflection period, a second written request must be submitted, which requires witnessing by two independent individuals. Finally, a third and final request is required prior to the prescription or administration of the lethal medication, thereby ensuring the patient's continued intent.

To address the potential deficiencies in the two-request system, it is necessary to increase the minimum number of requests to three as it would align the process with international best practices, establish a framework that incorporates an initial request, a formal written request, and a final request, thereby it will provide additional avenues for reflection. The introduction of mandatory waiting periods, for instance, between seven to fourteen days for each request, would allow for sustained deliberation and medical reassessment. Furthermore, requiring independent witnesses for the written request, explicitly ensuring that such requests are signed and witnessed by at least two individuals without financial or personal interest in the patient's decision, would bolster the integrity of the process.

Residency requirements and risk of medical tourism

The residency provisions outlined in the Assisted Dying Bill exhibit significant ambiguity, raising concerns about potential exploitation and the facilitation of medical tourism. Under the current framework, the absence of explicit requirements restricting access to assisted dying services to permanent residents or citizens of the UK introduces a legal loophole that could permit foreign nationals to travel to the UK specifically to access these services.

The lack of stringent residency requirements presents several potential risks, such as exploitation and abuse of the system, as individuals from jurisdictions where assisted dying is either illegal or heavily restricted could seek to access the provisions in the UK. This influx may overwhelm the healthcare system and divert resources from domestic patients, raising ethical concerns regarding the appropriateness of accommodating non-residents in such a deeply personal and medically intricate process. Furthermore, allowing non-residents to utilise assisted dying services can lead to the circumvention of legal and ethical safeguards [15]. Many countries have established prohibitive or stringent regulations about assisted dying; permitting non-residents to access services in the UK could encourage jurisdiction shopping, thereby undermining the integrity of both domestic and international regulations [16].

A comparative analysis of residency restrictions in other jurisdictions reveals that several places have acknowledged the risks associated with medical tourism in assisted dying and have implemented strict residency requirements. For instance, Switzerland permits assisted dying for non-residents; however, this practice has incited significant controversy and criticism relating to suicide tourism. In Canada, assisted dying was initially restricted to citizens and permanent residents, although legal challenges have prompted discussions about potential expansions, raising concerns about the impact on healthcare resources. In Oregon, individuals are required to provide proof of residency, such as a driver's license or tax records, to prevent non-residents from travelling specifically for assisted dying services. Similarly, in Victoria and Western Australia, explicitly limit access to those who have resided in these places for a minimum duration of 12 to 24 months prior to making a request.

The UK Parliament must implement strict residency requirements to mitigate the risks of misuse and uphold the Assisted Dying Bill's ethical integrity. Limiting access to UK citizens and permanent residents would ensure that assisted dying remains a service intended for individuals who possess demonstrable long-term ties to the UK, integrating them within the national healthcare system. Moreover, introducing a minimum period of continuous residence before eligibility is granted and mandating documentary proof of residency, such as UK passports, NHS registration records, and other forms of verified residency, would deter potential abuses of the system. Establishing a dedicated regulatory body to review residency claims and assess eligibility for assisted dying requests could bolster legal and medical oversight. Finally, implementing legal consequences for individuals who falsely claim residency to access assisted dying would promote accountability and deter fraudulent applications, thereby enhancing the ethical framework governing this sensitive issue.

Discrimination against patients with physical disabilities

The Assisted Dying Bill currently stipulates that terminally ill individuals must self-administer the prescribed lethal medication, provided that they possess the physical capability to do so. While this requirement aims to ensure voluntary participation and prevent coercion, it inadvertently discriminates against patients who suffer from severe physical disabilities, neuromuscular conditions, or other impairments that render them incapable of self-administration despite being mentally competent and fully informed regarding their decision. This stipulation creates an inequitable barrier, denying certain terminally ill individuals access to assisted dying solely based on their physical limitations rather than their cognitive or decisional capacity.

The requirement for self-administration disproportionately impacts individuals with specific conditions. For example, patients suffering from neuromuscular disorders, such as amyotrophic lateral sclerosis, multiple sclerosis, or muscular dystrophy, may experience progressive muscle weakness that prevents them from ingesting or handling the medication. Similarly, individuals with paralysis or advanced motor neuron disease who lack the physical ability to administer the medication independently face unjust exclusion from the process. Moreover, those with neurodegenerative conditions, such as Parkinson's disease or advanced multiple sclerosis, may experience severe tremors, muscle rigidity, or loss of coordination, rendering self-administration impractical or impossible. Additionally, many terminally ill patients experience extreme weakness and fatigue in the final stages of life, further hindering their ability to carry out the required actions for self-administration.

Denying access to assisted dying based solely on the criterion of physical ability raises significant ethical and legal concerns. This restriction constitutes a violation of equal access principles, as individuals who meet all legal and ethical requirements for assisted dying but are physically incapable of self-administration are unjustly discriminated. Furthermore, such a denial may result in undue suffering and a loss of autonomy, forcing individuals who cannot self-administer to endure prolonged suffering and depriving them of the right to a dignified death. The principles of informed consent are also compromised; the law acknowledges the autonomy of mentally competent individuals to make their own medical decisions, including the refusal of life-sustaining treatment. Consequently, excluding those who cannot self-administer from the process of assisted dying contradicts this fundamental principle. The inconsistencies between this regulation and other end-of-life practices become apparent. At the same time, the law allows healthcare professionals to administer palliative sedation in order to alleviate suffering in terminal patients. The prohibition of assistance in assisted dying for those physically unable to self-administer presents a contradiction.

Several jurisdictions have recognised this issue and implemented provisions allowing physician-assisted administration in cases where self-administration is not feasible. In Canada, the Medical Assistance in Dying legislation permits both self-administration and clinician-administered euthanasia, thereby ensuring equal access for individuals with physical disabilities [17]. Similarly, the Netherlands and Belgium primarily employ physician-administered euthanasia, avoiding discrimination based on physical capability. Although Switzerland requires self-administration in assisted suicide, it allows for flexibility in cases where physical limitations prevent independent action. Furthermore, regions such as Victoria and Western Australia, which initially mandated self-administration, have reviewed and revised their approaches to incorporate limited clinician-assisted options in specific circumstances.

To address this inequity and ensure that all eligible terminally ill individuals have access to assisted dying, several amendments to the Assisted Dying Bill are warranted. First, allowing clinician-assisted administration when medically necessary would enable healthcare practitioners, under strict guidelines, to assist in administering the lethal medication when a patient is physically incapable of doing so independently yet remains mentally competent [18-19]. Additionally, implementing safeguards to prevent abuse is crucial; assisted administration should be restricted to cases where an independent medical assessment confirms the patient's inability to self-administer and verifies that their request is both voluntary and free from coercion. Finally, a secondary approval process could be instituted to prevent misuse, requiring a second medical practitioner or an ethics panel to approve physician-assisted administration in situations where self-administration is not feasible. Such measures would promote equity and uphold the dignity of terminally ill individuals seeking assisted dying.

Need for a structured conscientious objection framework

The current iteration of the Assisted Dying Bill notably lacks explicit provisions regarding conscientious objection, which introduces potential inconsistencies in practice and could create unintended barriers to access for terminally ill individuals seeking assisted dying. While it is crucial to uphold the right of healthcare professionals to conscientiously object based on personal, ethical, or religious beliefs, the absence of a structured framework to manage these objections may disrupt care pathways, delay critical decision-making, and hinder patients' ability to access legally sanctioned services [20]. A significant lack of clarity exists concerning the scope of conscientious objection; without specific guidelines, uncertainties remain about whether such objections are limited to the direct administration of lethal medication or extend to providing information, referrals, or counselling services. This ambiguity may lead to inconsistent application of the law and confusion for both healthcare providers and patients alike [21].

Moreover, the unregulated environment surrounding conscientious objection could risk delayed or denied access to essential patient services. This concern is particularly acute for terminally ill individuals who possess a limited life expectancy, where timely access to assisted dying is imperative. Additionally, geographical disparities may emerge in service availability. In certain jurisdictions, a high concentration of objecting healthcare providers can result in significant geographical barriers, disproportionately affecting patients in rural or underserved areas. Implementing a structured framework promotes equitable access to these services irrespective of location.

Healthcare professionals who choose to object may also face ethical and legal uncertainties regarding their professional obligations. In the absence of a clearly defined policy, those who refuse participation may fear legal repercussions, while others could face professional sanctions for allegedly obstructing care. Internationally, several jurisdictions that have legalised assisted dying have proactively established structured frameworks that balance the rights of healthcare providers with those of patients. For instance, while healthcare providers reserve the right to refuse participation in Canada, they are legally mandated to refer patients to an alternative willing provider or a centralised coordination service, ensuring access while respecting professional autonomy. Similarly, in Victoria, the law permits healthcare professionals to object conscientiously but requires them to inform patients of available options for accessing non-objecting providers. In Switzerland and the Netherlands, measures exist to facilitate patient access to alternative providers without undue delays, even in cases where individual practitioners object.

The Assisted Dying Bill must incorporate a structured framework for conscientious objection to enhance ethical integrity and prioritise patient-centred care. This framework should explicitly define conscientious objection, clarifying whether it extends beyond direct involvement to encompass other aspects of patient care, such as providing information and referrals [22]. Moreover, a mandatory referral mechanism should be established, wherein healthcare professionals who object to assisting in dying must facilitate timely referrals to non-objecting providers or a centralised coordination service, ensuring that patients remain supported throughout the process. Establishing a national or regional body dedicated to coordinating assisted dying services would further aid patients in locating willing providers, thereby alleviating the burden on individual objectors and ensuring seamless access to care.

In addition, legal protections should be incorporated within the bill to shield healthcare providers who conscientiously object from discrimination, disciplinary actions, or potential termination of employment due to their refusal to participate. Furthermore, the bill should stipulate guidelines regarding institutional conscientious objection, particularly for healthcare facilities, such as faith-based hospitals, that may choose to prohibit assisted dying within their premises. Such guidelines should elucidate the extent to which institutions may object and outline the necessary protocols for facilitating patient transfers to complying facilities. Finally, ongoing education and ethical training opportunities must be provided for healthcare professionals to ensure they are well informed about their rights and responsibilities concerning conscientious objection, fostering a more structured and supportive environment for patients and practitioners.

Mandatory reporting as a safeguard against procedural breaches

One of the most significant omissions in the Assisted Dying Bill is the absence of a mandatory reporting mechanism designed to detect and address procedural breaches. Mandatory reporting has emerged as an essential tool for ensuring accountability, transparency, and compliance with ethical and legal standards in various regulatory and legal contexts, such as child protection and workplace safety. Implementing a similar framework within the assisted dying context would serve as a critical safeguard against violations, including coercion, improper medical assessments, procedural non-compliance, and ethical misconduct.

The process of assisted dying involves decisions that are often irreversible, rendering procedural integrity of utmost importance. The lack of a formal mechanism to identify breaches may allow errors in diagnosis, inadequate assessments of capacity, or procedural shortcuts to go undetected. A mandatory reporting system would establish a proactive oversight mechanism to ensure compliance with the legal and ethical standards articulated in the bill, thus preventing deviations that could undermine patient safety and autonomy. Furthermore, assisted dying remains a sensitive and controversial issue, and public confidence in the system relies heavily on stringent safeguards designed to prevent abuse or unethical practices. A robust reporting framework would enhance transparency and accountability, thereby reassuring the public that the law is being implemented responsibly and ethically [6].

Another compelling reason for mandatory reporting is the protection of vulnerable patients, including the elderly, disabled individuals, and those with limited access to healthcare. These populations may be susceptible to external pressures from family members, caregivers, or even healthcare professionals. By compelling those involved in the assisted dying process to report any suspected coercion or undue influence, a mechanism for independent investigations can be established, reinforcing the integrity of the process.

Moreover, the potential for medical misdiagnosis and errors in capacity assessment poses significant risks in the context of assisted dying. Misdiagnoses or failure to accurately assess decision-making capacity can lead to wrongful access to assisted dying. Mandatory reporting would require healthcare professionals to report suspected misdiagnoses, flawed prognoses, or questionable capacity assessments, facilitating corrective interventions before enacting irreversible decisions.

Additionally, the absence of clear guidelines surrounding reporting obligations can leave healthcare professionals uncertain regarding their responsibilities when they suspect breaches have occurred. Establishing a well-defined mandatory reporting protocol can alleviate ambiguity, thus protecting practitioners who come forward and ensuring they are not penalised for whistleblowing [23].

A review of international practices reveals that several jurisdictions with assisted dying legislation have instituted reporting requirements to uphold procedural integrity. For instance, in the Netherlands, physicians must report every assisted dying case to regional review committees, which assess compliance with legal standards. Any suspected violations are referred to legal authorities. Similarly, in Canada, the Medical Assistance in Dying legislation requires healthcare professionals to report cases to a federal oversight body, which monitors trends, ensures compliance, and identifies potential issues. In Victoria, the assisted dying framework incorporates a mandatory notification system where concerns related to non-compliance are reported to the Voluntary Assisted Dying Review Board.

To enhance the ethical and legal robustness of the assisted dying process, the legislation should mandate healthcare professionals, including but not limited to physicians, nurses, pharmacists, and mental health specialists, to report suspected breaches to a designated oversight body. These reports should address concerns such as coercion or undue influence on patients, procedural non-compliance (e.g., failure to meet eligibility criteria or bypassing required medical assessments), misdiagnoses leading to wrongful eligibility, and unethical conduct by healthcare professionals or third parties. Establishing an independent oversight and investigation body is essential to receiving and investigating such reports, conducting random audits of assisted dying cases, addressing complaints from medical professionals, family members, or other stakeholders, and imposing corrective actions. This regulatory entity should also have the authority to recommend legal action in cases of gross misconduct or violations of ethical standards.

Finally, legal protections for whistleblowers are paramount. Healthcare professionals should be safeguarded against retaliation, disciplinary actions, or legal consequences when reporting suspected breaches in good faith. Such protections create an environment where professionals feel empowered to uphold the integrity of the assisted dying process, leading to a practical and ethical framework foundation.

## Conclusions

While founded on principles of patient autonomy and dignity, the Assisted Dying Bill presents significant ethical, legal, and procedural shortcomings that necessitate urgent reform. The inadequacies in medical assessment and capacity determination expose patients to risks of misdiagnosis, undue influence, and flawed competency evaluations, highlighting the need for specialist involvement and stricter oversight. The insufficiency of the two-request system further compromises deliberative decision-making, emphasising the importance of extending the process to three requests with mandatory waiting periods. Additionally, the bill's lack of precise residency requirements raises concerns about medical tourism and resource allocation, warranting strict eligibility criteria to safeguard the system's integrity. Moreover, the requirement for self-administration unjustly discriminates against individuals with physical disabilities, contradicting the principles of equal access and informed consent. Adopting international best practices, such as independent psychiatric evaluations, strengthened procedural safeguards, and clinician-assisted options in cases of physical incapacity, would enhance the ethical and legal robustness of the legislation. Addressing these deficiencies is essential to ensuring that the Assisted Dying Bill protects vulnerable individuals while upholding autonomy and medical integrity.

A structured framework for conscientious objection and a mandatory reporting mechanism are essential to ensuring ethical integrity, transparency, and equitable access in implementing assisted dying legislation. Without clear guidelines on conscientious objection, inconsistencies in practice may lead to care disruptions, delays, and geographical disparities in service availability. Implementing a structured framework that mandates timely referrals, establishes a central coordination body, and provides legal protections for objecting healthcare professionals would balance practitioners' rights with patients' needs. Similarly, the absence of a mandatory reporting system leaves the assisted dying process vulnerable to procedural breaches, coercion, and medical errors. Introducing a robust reporting framework would enhance accountability, protect vulnerable populations, and uphold public confidence in the ethical application of assisted dying laws. An independent oversight body, alongside legal protections for whistleblowers, would further reinforce procedural safeguards and deter misconduct. By integrating these measures, the Assisted Dying Bill can achieve a more comprehensive, ethically sound, and patient-centred approach, ensuring that both the rights of healthcare providers and the needs of terminally ill patients are adequately addressed.
